# Proton Pump Inhibitor-Induced Gut Dysbiosis Increases Mortality Rates for Patients with Clostridioides difficile Infection

**DOI:** 10.1128/spectrum.00486-22

**Published:** 2022-07-06

**Authors:** Cheng-Yu Lin, Hao-Tsai Cheng, Chia-Jung Kuo, Yun-Shien Lee, Chang-Mu Sung, Micah Keidan, Krishna Rao, John Y. Kao, Sen-Yung Hsieh

**Affiliations:** a Department of Gastroenterology and Hepatology, Chang Gung Memorial Hospital, Taoyuan, Taiwan; b Chang Gung University College of Medicine, Taoyuan, Taiwan; c Department of Gastroenterology and Hepatology, Tu Cheng Hospital, New Taipei City, Taiwan; d Department of Biotechnology, Ming Chuan University, Taoyuan, Taiwan; e Department of Internal Medicine, Division of Infectious Diseases, Michigan Medicine, University of Michigan, Ann Arbor, Michigan, USA; f Department of Internal Medicine, Division of Gastroenterology, Michigan Medicine, University of Michigan, Ann Arbor, Michigan, USA; Wayne State University

**Keywords:** CDI-associated gut dysbiosis, PPI-induced gut dysbiosis, gut microbiota

## Abstract

Clostridioides difficile infection (CDI) is associated with high mortality rates among patients with chronic illnesses. We aimed to identify avoidable risk factors to reduce the mortality rate in CDI patients. A total of 306 patients with diarrhea and clinical suspicion of CDI were enrolled, and fecal samples were gathered from 145 patients. CDI was diagnosed by fecal positivity for the C. difficile *tcdB* gene. Risk factors associated with death within 180 days were identified using Cox regression analysis. The fecal microbiota was determined through bacterial 16S rRNA gene sequencing. Of the patients with diarrhea, 240 (mean age, 69.1 years) were positive for CDI, and 91 died within 180 days. Multivariate analysis revealed that male sex, high Charlson Comorbidity Index and McCabe scores, high serum C-reactive protein levels, low hematocrit levels, low absolute eosinophil counts, high neutrophil/lymphocyte ratios, and daily use of proton pump inhibitors (PPIs) were independent risk factors for overall mortality. Cumulative analyses confirmed the association of duration-dependent PPI use with a high mortality rate. Fecal microbiota analyses showed associations of decreased relative abundance of Ruminococcus gnavus (*P* = 0.001) and Prevotella copri (*P* = 0.025) and increased relative abundance of Parabacteroides merdae (*P* = 0.001) and Clostridioides difficile (*P* = 0.040) with higher mortality rates in patients with CDI. Moreover, these microbiota changes were correlated with the duration of PPI use.

**IMPORTANCE** This article demonstrates that daily PPI use was the only avoidable risk factor for death. With more extended PPI use, the mortality rate was higher in patients with CDI. Decreases in Prevotella copri and Ruminococcus gnavus and increases in Parabacteroides merdae and Clostridioides difficile in line with daily PPI use duration were significantly associated with the death of CDI patients. Our findings provide in-depth insights into the cautious use of PPIs in chronically ill patients with CDI.

## INTRODUCTION

Clostridioides difficile is an anaerobic, spore-forming, toxin-producing, Gram-positive bacterium. Clostridioides difficile infection (CDI) is one of the most common causes of antibiotic-associated diarrhea (1) and health care-associated infections, particularly in aged or chronically ill patients (2). CDI is characterized by diverse clinical presentations, from mild diarrhea to fulminant toxic colitis. CDI advances severity and dramatically increases mortality rates for patients with acute and chronic illnesses. A previous study in Europe revealed that up to 23% of all-cause hospital deaths were attributable to CDI (3). A recent retrospective nationwide cohort study showed that CDI was associated with a 1.77- to 2.52-fold increased risk of 30-day all-cause death in the propensity-score-matched-pairs analysis (4), CDI significantly impacts long-term clinical outcomes in aged patients, contributing to mortality rates of 14.9% and 19.2% at 60 days and 1 year, respectively (5). Because the global population is aging and chronic diseases and hospitalizations are increasing, there is an unmet need to identify manageable risk factors and avoid them to reduce CDI-associated death.

Proton pump inhibitors (PPIs) are irreversible inhibitors of gastric H^+^/K^+^-ATPase in parietal cells to reduce acid secretion. The degree and duration of gastric hypoacidity with PPIs exceed the effects of histamine-2 receptor antagonists. Due to their high levels of effectiveness and safety in the amelioration of gastroesophageal reflux disease and the treatment and prevention of gastrointestinal ulcers and bleeding, PPIs have become the medications for elderly and chronically ill patients that are most prescribed by health care providers. However, accumulating evidence suggests an association between PPI use and various adverse effects (6), including CDI (7–11) and gut dysbiosis (12–15). Therefore, it is essential to clarify the safety of PPI use in chronically ill patients, particularly those with CDI.

Here, we report our study to identify manageable risk factors associated with high mortality rates among adult patients with CDI. We found that PPI was an independent risk factor for death, in a dose-dependent manner. We further identified a correlation between PPI-associated gut dysbiosis and death among patients with CDI. Our findings propose caution for PPIs in patients with CDI, particularly those with chronic illnesses.

## RESULTS

### Patients and study design.

**FIG 1:**
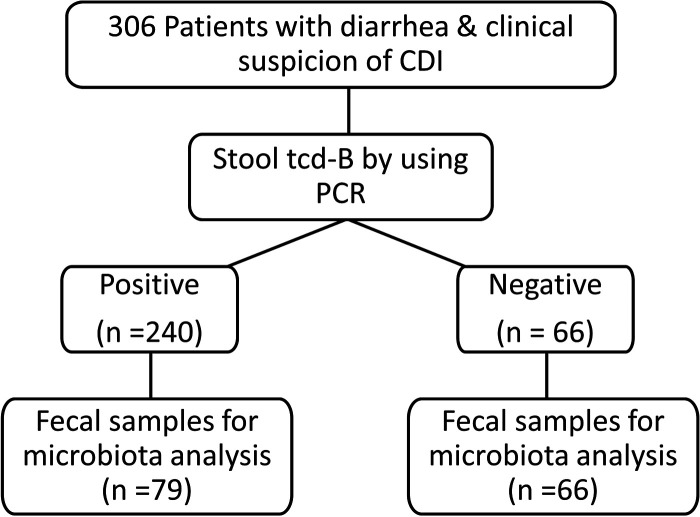
Patient selection. A total of 306 patients with diarrhea who underwent stool tests for CDI through PCR-based detection of the toxin B gene (*tcdB*) of C. difficile were included in the study from August 2016 to July 2018. Fecal samples were collected from July 2017 and were available for only 145 consequential cases.

We enrolled 306 consecutive diarrhea patients who were clinically suspected to have CDI and subjected them to PCR testing for *tcdB* (Fig. 1). Of these, 240 cases were positive for *tcdB* and diagnosed with CDI. According to the disease onset, 166 (69.2%), 41 (17.1%), 11 (0.4%), and 22 (9.2%) cases were categorized as health care facility-onset health care facility-associated (HO-HCFA), community-onset health care facility-associated (CO-HCFA), indeterminate onset (ID), and community-associated (CA) CDI, respectively. As shown in Table 1, there was no statistical difference between patients with versus without CDI in terms of clinical characteristics, including age (mean age of 69 years in both groups), gender (48.8% versus 51.5%), comorbidities (including diabetes mellitus, chronic renal diseases, solid cancers, and hematological malignancies), Charlson Comorbidity Index and McCabe scores, main laboratory data, exposure to antibiotics or PPIs, and cumulative rates of death within 180 days (37.9% versus 30.3%).

**TABLE 1:** Demographic characteristics of diarrhea patients with and without CDI

Parameter^*a*^	Data for patients with:		*P*
	CDI diarrhea (*N* = 240)	Non-CDI diarrhea (*N* = 66)	
Age (mean ± SD) (yr)	69.1 ± 17.0	69.0 ± 16.9	0.99
Gender (no. [%])			0.78
Male	117 (48.8)	34 (51.5)	
Female	113 (50.2)	32 (48.5)	
Charlson Comorbidity Index score (mean ± SD)	6.43 ± 3.30	6.18 ± 3.31	0.60
Comorbidity (no. [%])			
Diabetes mellitus	91 (37.9)	24 (36.4)	0.89
Diabetes with end organ damage	60 (25.0)	10 (15.2)	0.10
Chronic kidney disease	57 (23.8)	19 (28.8)	0.42
Hematological cancer	26 (10.8)	2 (3.0)	0.06
Solid organ cancer	60 (25)	22 (33.3)	0.21
Metastatic solid organ cancer	34 (14.2)	11 (16.7)	0.70
History of cerebral vascular accident	42 (17.5)	15 (22.7)	0.37
Connective tissue disorder	21 (8.8)	5 (7.6)	1.00
Liver disease	52 (21.7)	12 (18.2)	0.61
Portal hypertension	13 (5.4)	4 (6.1)	0.77
Hemiplegia	37 (15.4)	4 (6.1)	0.06
PAOD	16 (6.7)	2 (3.0)	0.38
History of myocardial infarction	21 (8.8)	2 (3.0)	0.19
Dementia	20 (8.3)	7 (10.6)	0.62
Congestive heart failure	27 (11.3)	5 (7.6)	0.50
COPD	17 (7.1)	3 (4.5)	0.58
Recent peptic ulcer disease^*b*^	24 (10.0)	2 (3.0)	0.08
McCabe score (no. [%])			0.91
0 or 1	205 (85.4)	56 (84.8)	
2	35 (14.6)	10 (15.2)	
Critical illness (yes) (no. [%])	53 (22.1)	13 (19.7)	0.74
Antibiotic exposure (yes) (no. [%])^*c*^	213 (88.8)	59 (89.4)	1.00
Duration of PPI use (mean ± SD) (days)	7.8 ± 10.3	6.3 ± 9.9	0.28
Parenteral nutrition (yes) (no. [%])	19 (7.9)	3 (4.5)	0.43
Creatinine level (mean ± SD) (μmol/L)	169.7 ± 198.8	159.1 ± 173.0	0.70
CRP level (mean ± SD) (mg/L)	82.8 ± 75.4	73.8 ± 85.0	0.42
Hematocrit level (mean ± SD) (%)	30.1 ± 5.6	30.3 ± 5.4	0.79
Platelet count (mean ± SD) (10^9^ cells/L)	202.5 ± 136.2	193.8 ± 96.8	0.57
WBC count (mean ± SD) (10^9^ cells/L)	10.0 ± 6.0	9.4 ± 5.3	0.49
AEC (mean ± SD) (10^9^ cells/L)	0.14 ± 0.20	0.21 ± 0.49	0.27
NLR (mean ± SD)	9.7 ± 11.7	12.0 ± 15.5	0.19
Death within 90 days (no. [%])	72 (30.0)	18 (27.3)	0.76
Death within 180 days (no. [%])	91 (37.9)	20 (30.3)	0.31

a PAOD, peripheral arterial occlusive disease; COPD, chronic obstructive pulmonary disease; CRP, C-reactive protein; AEC, absolute eosinophil count; WBC, white blood cell; NLR, neutrophil/lymphocyte ratio.

b Endoscopically proven peptic ulcer disease within 3 months before the index date.

c Antibiotic exposure within 28 days before the index date.

### Factors associated with death for patients with CDI.

We used Cox regression analyses to elucidate the factors associated with death among patients with CDI (Table 2). Multivariate analyses revealed that male sex (adjusted hazard ratio [aHR], 1.73 [95% confidence interval [CI], 1.06 to 2.82]), HO-HCFA CDI (aHR, 2.64 [95% CI, 1.41 to 4.93]), high Charlson Comorbidity Index score (aHR, 1.18 [95% CI, 1.09 to 1.28]) or McCabe score (aHR, 2.39 [95% CI, 1.30 to 4.39]), high C-reactive protein level (aHR, 1.01 [95% CI, 1.00 to 1.01]), low hematocrit level (aHR, 0.92 [95% CI, 0.88 to 0.97]), low absolute eosinophil count (aHR, 0.14 [95% CI, 0.03 to 0.61]), high neutrophil/lymphocyte ratio (aHR, 1.03 [95% CI, 1.01 to 1.04]), and daily PPI use (aHR, 1.20 per week [95% CI, 1.01 to 1.43]) were associated with high mortality rates among CDI patients. For individual comorbidity diseases, multivariate analyses revealed that daily PPI use (aHR, 1.23 per week [95% CI, 1.08 to 1.40]), chronic kidney disease (aHR, 2.30 [95% CI, 1.45 to 3.62]), hematological cancer (aHR, 3.47 [95% CI, 2.01 to 6.00]), metastatic solid cancer (aHR, 3.02 [95% CI, 1.52 to 6.01]), peripheral arterial occlusive disease (aHR, 2.82 [95% CI, 1.43 to 5.59]), and chronic obstructive pulmonary disease (aHR, 2.35 [95% CI, 1.25 to 4.42]) (see Table S1 in the supplemental material) were also independent risk factors associated with death for patients with CDI.

**TABLE 2:** Cox regression analyses of hazard factors associated with death within 180 days

Parameter^*a*^	Univariate analysis		Multivariate analysis	
	HR	*P*	HR (95% CI)	*P*
Age (per yr)	1.014	0.04	0.994 (0.978–1.012)	0.53
Gender				
Female	Ref^*b*^		Ref	
Male	1.499	0.06	1.725 (1.055–2.819)	0.03
CDI exposure				
Other than HO-HCFA	Ref		Ref	
HO-HCFA	2.332	<0.01	2.636 (1.411–4.926)	<0.01
Charlson Comorbidity Index score	1.194	<0.01	1.180 (1.088–1.279)	<0.01
McCabe score				
0 or 1	Ref		Ref	
2	3.069	<0.01	2.394 (1.304–4.393)	0.01
Critical illness status	1.963	<0.01	0.916 (0.526–1.592)	0.76
Antibiotic exposure	3.414	0.02	1.403 (0.464–4.243)	0.55
PPI (per wk)	1.287	<0.01	1.198 (1.007–1.426)	0.04
Parenteral nutrition	2.038	0.02	1.196 (0.587–2.437)	0.62
Creatinine level	1.001	0.19	1.000 (0.998–1.001)	0.51
CRP level	1.006	<0.01	1.005 (1.002–1.007)	<0.01
Hematocrit level	0.928	<0.01	0.924 (0.876–0.973)	<0.01
Platelet count	0.997	<0.01	0.998 (0.996–1.001)	0.14
WBC count	1.005	0.79	1.029 (0.981–1.080)	0.24
AEC	0.253	0.05	0.144 (0.034–0.610)	<0.01
NLR	1.015	0.02	1.026 (1.010–1.043)	<0.01

a CRP, C-reactive protein; WBC, white blood cell; AEC, absolute eosinophil count; NLR, neutrophil/lymphocyte ratio.

b Ref, reference.

### Duration of daily PPI use is positively correlated with CDI mortality rates.

**FIG 2:**
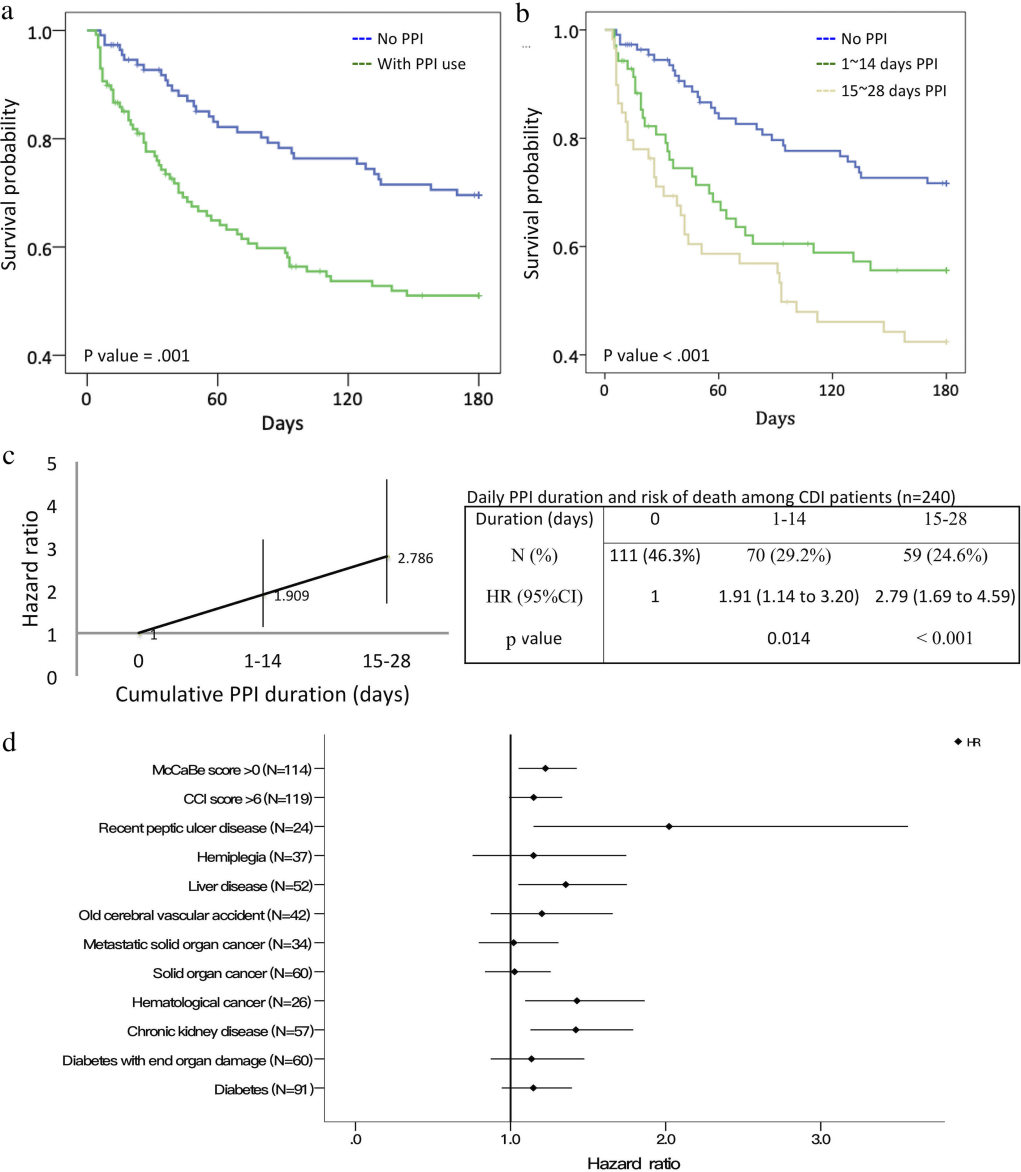
Survival analyses of CDI patients with or without daily PPI treatment. (a) The CDI patients were divided into patients with or without PPI treatment. (b) The patients with PPI treatment were further divided based on continuous daily PPI use for up to 14 days (*n* = 70) or for 15 to 28 days (*n* = 59). (c) Relationship between the duration of PPI use and the HR for overall mortality. (d) The CDI patients were further divided based on different underlying comorbid disorders.

Of the independent risk factors for CDI-related death, we mainly focused on medical management since patient survival could be improved by avoiding such management in clinical practice. Daily PPI use was the only independent risk factor associated with a high mortality rate (Table 2). Kaplan-Meier survival curves further demonstrated that patients with PPI use had a higher cumulative mortality rate (*P* = 0.001, log rank test) (Fig. 2a), and the association was duration dependent (*P* < 0.001, log rank test) (Fig. 2b). More importantly, there was a positive relationship between the duration of daily use of PPIs and the cumulative rate of death within 180 days (daily PPI use for 1 to 14 days: hazard ratio [HR], 1.91 [*P* = 0.014]; daily PPI use for 15 to 28 days: HR, 2.79 [*P* < 0.001]) (Fig. 2c). Together, PPI use was a dose-dependent risk factor for death among patients with CDI.

Stratified Cox regression analysis was performed to verify the association of PPI use with death. As shown in Fig. 2d, daily PPI use was significantly associated with 180-day mortality rates for patients with chronic kidney disease (HR, 1.421 [95% CI, 1.128 to 1.791]), hematological cancer (HR, 1.428 [95% CI, 1.094 to 1.865]), or recent peptic ulcer disease (HR, 2.022 [95% CI, 1.148 to 3.563]) but not for those with solid organ cancer (HR, 1.026 [95% CI, 0.835 to 1.259]) or diabetes (HR, 1.147 [95% CI, 0.943 to 1.396]).

### PPI alters the gut microbiome in patients with CDI.

**FIG 3:**
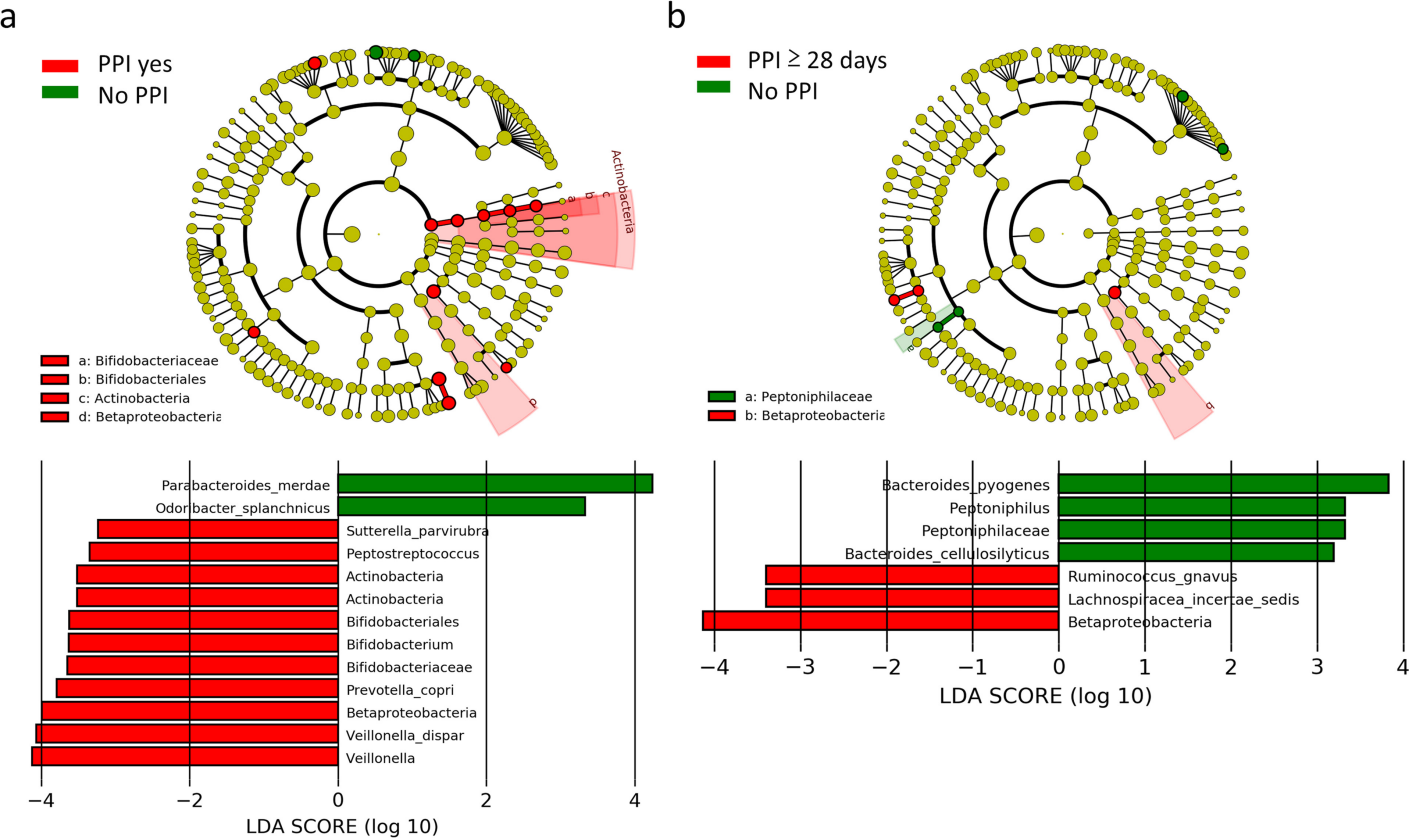
Cladograms for differentially abundant taxa in the gut microbiota. Taxonomic cladograms using LEfSe analysis show the most differentially abundant taxa between CDI patients with versus without daily PPI use (a) and between CDI patients with prolonged PPI treatment (≥28 days) and untreated patients (b). Notably, the taxa with mean abundance of <0.1% were excluded from the analyses. The results of linear discriminant analysis (LDA) are shown at the bottom. Only taxa meeting an LDA threshold of >2.0 are shown.

Given that prolonged PPI use may alter the gut microbiome (12–14, 16), we speculated that PPI-induced gut dysbiosis contributed to mortality rates for patients with CDI. We compared the gut microbiomes of CDI patients with versus without PPI use. After excluding taxa with relative abundance of <0.1%, we found that daily PPI use led to a decrease in the relative abundance of Prevotella copri and Veillonella dispar and an increase in that of Parabacteroides merdae and Odoribacter splanchnicus (Fig. 3a and Table 3; also see Table S2). Prolonged PPI use (≥28 days) further decreased the relative abundance of Ruminococcus gnavus (phylum *Firmicutes*, family *Lachnospiraceae*) and increased that of Bacteroides pyogenes and Bacteroides cellulosilyticus (Fig. 3b and Table 3; also see Table S3).

**TABLE 3:** Gut microbes with significantly different abundances in CDI patients with versus without daily PPI use^*a*^

Comparison and microbe	Relative abundance in:						PPI use/no PPI use ratio	PPI use for ≥28 days/no PPI use ratio	*P*
	Non-PPI-treated patients (*N* = 29)		PPI users (*N* = 50)		Patients with prolonged PPI use (*N* = 12)				
	Mean	SD	Mean	SD	Mean	SD			
Daily PPI users vs untreated patients									
Parabacteroides merdae	0.0105	0.0218	0.0473	0.0754			4.49		0.05
Odoribacter splanchnicus	0.0009	0.0035	0.0051	0.0095			5.42		0.05
*Peptostreptococcus*	0.0052	0.0227	0.0009	0.0041			0.18		<0.01
*Bifidobacterium*	0.0070	0.0198	0.0011	0.0039			0.15		0.02
*Bifidobacteriales* order	0.0070	0.0198	0.0011	0.0039			0.16		0.03
*Actinobacteria* class	0.0117	0.0313	0.0046	0.0160			0.40		0.05
Prevotella copri	0.0173	0.0547	0.0144	0.0599			0.83		0.04
*Betaproteobacteria* class	0.0414	0.0584	0.0183	0.0287			0.44		0.05
Veillonella dispar	0.0417	0.0590	0.0212	0.0771			0.51		0.02
*Veillonella*	0.0450	0.0641	0.0214	0.0774			0.48		0.02
Patients with daily PPI use for ≥28 days vs untreated patients									
Bacteroides pyogenes	<1.0E−07	<1.0E−07			0.0132	0.0458		NA^*b*^	0.03
Bacteroides cellulosilyticus	0.0015	0.0035			0.0052	0.0055		3.34	0.04
*Peptoniphilus*	0.0001	0.0002			0.0021	0.0072		43.74	0.04
*Lachnospiracea incertae sedis*	0.0057	0.0170			0.0046	0.0160		0.80	0.01
Ruminococcus gnavus	0.0056	0.0170			0.0046	0.0160		0.82	0.04
*Betaproteobacteria* class	0.0414	0.0585			0.0122	0.0163		0.30	0.05

a Total amount of all the taxa in each individual was normalized to be 1.0000. Taxa with mean relative abundances of <0.1% or *P* values of >0.05 were excluded.

b NA, not applicable.

### PPI-induced gut dysbiosis is associated with high mortality rates for patients with CDI.

**FIG 4:**
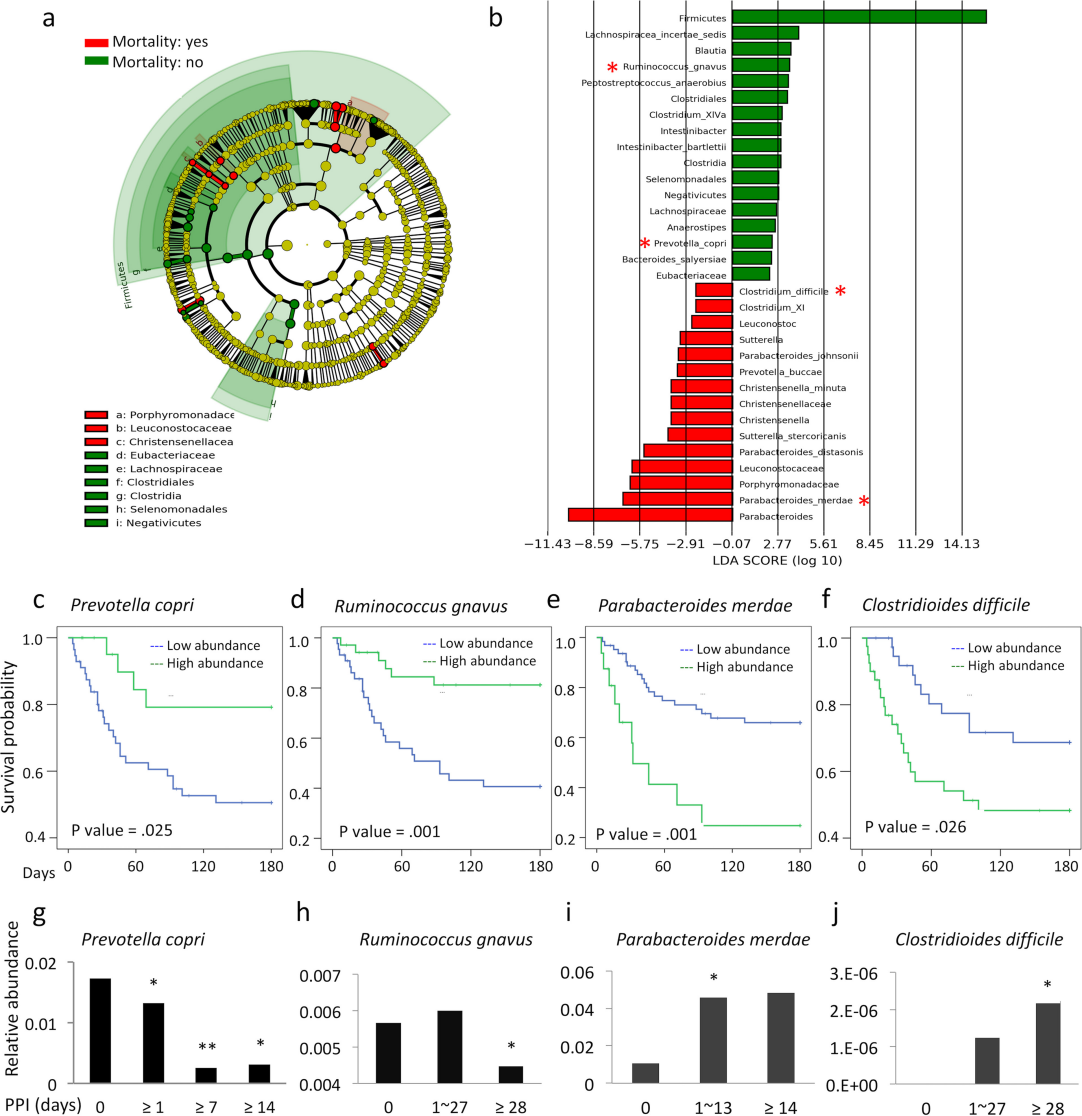
PPI-associated gut dysbiosis related to death among patients with CDI. (a) Cladogram of the differentially abundant taxa of the gut microbiota between CDI patients with versus without death within 180 days. (b) Only taxa meeting an LDA threshold of >2.0 are shown. (c to f) Kaplan-Meier curves for cumulative survival times for patients with high (green) and low (blue) relative abundance of Prevotella copri (c), Ruminococcus gnavus (d), *Parabaceroides merdae* (e), and Clostridioides difficile (f). *P* values were measured using the log rank test. (g to j) Correlation of the relative abundance with the duration of daily PPI use for Prevotella copri (g), Ruminococcus gnavus (h), *Parabaceroides merdae* (i), and Clostridioides difficile (j). *, *P* < 0.05; **, *P* < 0.01, compared with the mean levels among patients without PPI. Notably, panel j was derived from the relative abundance of Clostridioides difficile in patients with negative *tcdB* test results. The results were unable to establish the relationship between the duration of PPI use and the fecal abundance of Clostridioides difficile because of its high level of abundance in patients with CDI.

We then determined whether PPI-induced gut dysbiosis was associated with death among patients with CDI. We compared the gut microbiomes of CDI patients with versus without death. After exclusion of taxa with relative abundance of <0.1%, the decrease in the relative abundance of *Ruminoccous gnavus* and Prevotella copri and the increase in the relative abundance of genus *Parabacteroides*, including Parabacteroides merdae, and Clostridioides difficile were associated with death among patients with CDI (Fig. 4a and b). Kaplan-Meier curves further confirmed that low relative abundance of Prevotella copri (*P* = 0.025) (Fig. 4c) and Ruminococcus gnavus (*P* = 0.001) (Fig. 4d) and high relative abundance of Parabacteroides merdae (*P* = 0.001) (Fig. 4e) and Clostridioides difficile (*P* = 0.026) (Fig. 4f) were associated with low survival rates for patients with CDI. Cox regression analysis further confirmed that the relative abundance of Prevotella copri (HR, 0.41 [95% CI, 0.192 to 0.887]; *P* = 0.04) and Ruminococcus gnavus (HR, 0.29 [95% CI, 0.139 to 0.586]; *P* < 0.01) was inversely related to mortality rates for patients with CDI, whereas that of Parabacteroides merdae (HR, 6.77 [95% CI, 2.284 to 20.072]; *P* < 0.01) and Clostridioides difficile (HR, 2.28 [95% CI, 1.105 to 4.711]; *P* = 0.04) was positively associated with mortality rates for patients with CDI (Table 4). We further correlated the relative abundance of Clostridioides difficile with that of Parabacteroides merdae, Prevotella copri, and Ruminococcus gnavus. We found that the relative abundance of Prevotella copri, but not others, had a significant correlation with Clostridioides difficile (Spearman’s rho = −0.315; *P* = 0.005) (see Table S4). On the other hand, multivariate analyses revealed that Ruminococcus gnavus (aHR, 0.29 [95% CI, 0.11 to 0.75]) and Parabacteroides merdae (aHR, 2.94 [95% CI, 1.27 to 6.78]) were significantly associated with high mortality rates for CDI patients (see Fig. S1). These findings further supported the independent roles of Ruminococcus gnavus and Parabacteroides merdae in the clinical outcomes of CDI patients.

**TABLE 4:** Gut microbes with significant differences in relative abundance in CDI patients with versus without death within 180 days^*a*^

Microbe	Relative abundance among patients who:				Overall mortality	
	Survived		Died			
	Mean	SD	Mean	SD	HR	*P*
*Parabacteroides*	0.0366	0.0479	0.0883	0.1016	10.14	<0.01
Parabacteroides merdae	0.0197	0.0386	0.0557	0.0864	6.77	<0.01
*Porphyromonadaceae* family	0.0618	0.0704	0.1290	0.1241	6.33	<0.01
*Leuconostocaceae* family	0.0038	0.0194	0.0013	0.0046	6.22	<0.01
Parabacteroides distasonis	0.0088	0.0185	0.0193	0.0315	5.49	<0.01
Sutterella stercoricanis	0.0011	0.0073	0.0012	0.0051	4.00	0.04
Christensenella minuta	<0.0001	0.0001	0.0011	0.0054	3.82	0.02
*Christensenella*	<0.0001	0.0001	0.0011	0.0054	3.82	0.02
*Christensenellaceae* family	<0.0001	0.0001	0.0011	0.0054	3.82	0.02
Prevotella buccae	0.0058	0.0271	0.0003	0.0013	3.42	0.02
*Sutterella*	0.0026	0.0102	0.0025	0.0059	3.24	0.04
*Leuconostoc*	0.0034	0.0192	0.0004	0.0012	2.53	0.04
Clostridioides difficile	0.0152	0.0267	0.0243	0.0411	2.28	0.04
*Clostridium XI*	0.0152	0.0267	0.0243	0.0411	2.28	0.04
*Eubacteriaceae* family	0.0029	0.0048	0.0013	0.0033	0.44	0.05
Bacteroides salyersiae	0.0018	0.0046	0.0006	0.0016	0.42	0.04
Prevotella copri	0.0187	0.0665	0.0103	0.0398	0.41	0.04
*Lachnospiraceae* family	0.0655	0.0484	0.0493	0.0528	0.37	0.02
*Selenomonadales* order	0.0732	0.0653	0.0503	0.0979	0.35	0.02
*Negativicutes* class	0.0732	0.0653	0.0503	0.0979	0.35	0.02
*Clostridia* class	0.1800	0.1120	0.1236	0.0987	0.34	0.01
*Clostridium* XlVa	0.0304	0.0275	0.0265	0.0387	0.33	0.02
*Clostridiales* order	0.1708	0.1150	0.1185	0.0941	0.30	<0.01
Peptostreptococcus anaerobius	0.0031	0.0169	<0.0001	<0.0001	0.29	0.02
Ruminococcus gnavus	0.0079	0.0168	0.0020	0.0058	0.29	<0.01
*Blautia*	0.0040	0.0082	0.0011	0.0035	0.28	<0.01
*Lachnospiracea incertae sedis*	0.0080	0.0169	0.0020	0.0058	0.25	<0.01
*Firmicutes* phylum	0.3109	0.1417	0.2421	0.1939	0.06	<0.01

a Total amount of all the taxa in each individual was normalized to be 1.0000. Taxa with mean relative abundances of <0.1% or *P* values of >0.05 were excluded.

### Death-associated gut dysbiosis is correlated with the duration of PPI use.

We further found that there was a negative correlation between the relative levels of Prevotella copri and Ruminococcus gnavus and the duration of PPI use (Fig. 4g and h), while there was a positive correlation between the relative abundance of *Parabacteriodes merdae* and Clostridioides difficile and the period of PPI use (Fig. 4i and j). The results further support the causative role of prolonged PPI use in gut dysbiosis-related death among patients with CDI.

Notably, our findings of a dose-dependent association of daily PPI use with the relative abundance of fecal Clostridioides difficile (Fig. 4j) and a positive correlation between the abundance of fecal Clostridioides difficile and high mortality rates (Fig. 4f) further strengthen the impact of prolonged PPI use on the induction and aggravation of the severity of CDI in patients with chronic illnesses.

## DISCUSSION

Recently, there have been concerns regarding the increased incidence of CDI among PPI users (7–11). CDI in chronically ill patients is a severe infection with high mortality rates (17). Therefore, it is crucial to clarify the safety of PPI use in chronically ill patients, particularly those with CDI. This study showed a duration-dependent association between daily PPI use and mortality rates for patients with CDI. This finding is in line with a previous report suggesting that PPI exposure is an independent risk factor for severe outcomes from CDI (18). Our findings are of clinical significance because PPI use is avoidable and manageable. We demonstrated PPI-associated gut dysbiosis and its association with CDI mortality rates. It is less likely that the association of PPI use with mortality rates for CDI patients is related to the indication for PPI use, such as a peptic ulcer or malignancy, because stratified Cox regression analyses revealed that PPI use was a risk factor for death in each subgroup of CDI patients with an individual comorbid disease, including peptic ulcer disease, solid cancer, and hematological malignancy. Moreover, the inverse correlations of the duration of PPI use with the time to survival in CDI patients and with the changes in relative abundance with the death-associated gut dysbiosis further support our hypothesis that PPI increases the mortality rates for CDI patients by inducing gut dysbiosis. Interestingly, prolonged daily PPI use was correlated with an increase in the relative abundance of fecal C. difficile, suggesting that prolonged PPI use might precipitate the incidence of CDI recurrence. Although our cohort did not reveal such an association, our findings deserve further studies with a larger cohort and a longer follow-up period.

Indeed, clinical restoration to gut eubiosis by fecal microbiota transplantation (FMT) has successfully treated recurrent and refractory CDI (19–21). We further identified the microbes involved in PPI-associated gut dysbiosis and their levels, including decreases in the abundance of Prevotella copri, *Lachinospiraceae*, and Ruminococcus gnavus and increases in the levels of Parabacteroides merdae and Clostridioides difficile, which contributed to high mortality rates in patients with CDI. We speculate that targeting PPI-associated gut dysbiosis by avoiding PPI use would decrease the mortality rates for acute or chronically ill patients with CDI. Indeed, a recent study showed that coadministration of probiotics to adults and children prescribed antibiotics was associated with a lower risk of CDI (22). Our findings support further prospective studies to validate the benefits of probiotics in reducing CDI incidence and mortality rates for chronically ill patients receiving antibiotic treatments.

The relative abundance of Parabacteroides merdae was increased in PPI users and was associated with a high mortality rate in patients with CDI. Parabacteroides merdae is a Gram-negative anaerobic microbe, and it commonly colonizes the gastrointestinal tract. Recent studies revealed the involvement of Parabacteroides merdae in various human diseases, such as hypertension (increased abundance) (23), colon cancer (colonization in tumor tissues) (24), ulcerative colitis (decreased abundance) (25), and acute-on-chronic liver failure in patients with chronic hepatitis B (26). In addition, Parabacteroides merdae secretes PmC11, a monomeric cysteine protease that is topologically similar to the cysteine protease domains of Clostridioides difficile toxins (27, 28). It is speculated that PmC11 of Parabacteroides merdae may synergistically aggravate the cytotoxicity induced by C. difficile toxins.

*Prevotella*, a Gram-negative, anaerobic, and non-spore-forming bacillus, is one of the dominant genera in *Bacteroidetes* and is commonly enriched among individuals who consume a plant-rich diet. In the human microbiome, *Prevotella* species are highly abundant and play critical roles in balancing health and diseases (29). Although increased Prevotella copri levels have been shown to exacerbate chronic inflammation in rheumatoid arthritis (30–32), Prevotella copri has been reported to increase glucose tolerance and enhance insulin resistance. A decrease in its abundance is associated with type 2 diabetes and ischemic cardiovascular disease (33). Prevotella copri, one of the primary inhabitants of the human gut, plays a pivotal role in maintaining the homeostasis of the host immune system and metabolism. Prolonged PPI use leads to a decrease in gut Prevotella copri abundance, which may perturb normal immunity and metabolism and worsen clinical outcomes for patients with CDI.

Ruminococcus gnavus belongs to the family *Lachnospiraceae*, which comprises anaerobic and spore-forming bacteria. Although aberrant increases in its levels in the gut are associated with inflammatory diseases, such as inflammatory bowel disease, spondyloarthritis, and infantile eczema, probably via the production of proinflammatory polysaccharides (34, 35), *in vitro* studies revealed that Ruminococcus gnavus produces the antimicrobial peptides ruminococcin A and ruminococcin C1 (36), with activity against C. difficile and clinical potential for anti-CDI treatments. In addition, the members of *Lachinospiraceae* ferment dietary fiber in the gut into short-chain fatty acids (SCFAs), such as acetate, propionate, and butyrate (37). The SCFAs are a part of the energy sources of colonic epithelial cells, maintain intestinal barrier integrity (the bowel barrier), and protect against intestinal inflammation (38). The abundance of bacteria that ferment fibers to SCFAs is typically reduced in the mucosa and feces of patients with inflammatory bowel disease, compared with healthy individuals (39). SCFAs regulate the size and function of the colonic pool of regulatory T cells to protect against colitis and promote colonic homeostasis and health (40) by increasing the expression of antimicrobial peptides and modulating the production of immune mediators for the repair and maintenance of epithelial integrity. SCFAs can also regulate the differentiation, recruitment, and activation of neutrophils, dendritic cells, macrophages, and T lymphocytes (41). Moreover, a recent study revealed the restoration of SCFAs following FMT in patients with recurrent CDI (42).

### Conclusions.

Overall, the gut microbiome of a healthy individual is a balanced community containing diverse microbes that maintain local and systemic homeostasis, including intestinal mucosa integrity, immune responses, and metabolism, between the host and microbes. Prolonged PPI use perturbs the gut microbiome and disrupts homeostasis, resulting in deterioration and death, particularly among chronically ill patients. Our findings provide a rationale for the cautious use of PPIs in patients with CDI and other chronic illnesses.

## MATERIALS AND METHODS

After exclusion of patients with previous CDI episodes or age of <18 years, a total of 306 diarrhea patients with clinical suspicion of CDI who had fecal C. difficile *tcdB* tests (BD Max Cdiff nucleic acid amplification test) during the period from August 2016 to July 2018 were enrolled in this study. Diarrhea was defined as at least three loose or watery stool passages within 24 h for at least 2 consecutive days. Seventy-nine and 66 fecal samples, which belonged to the group with a final diagnosis of CDI and the non-CDI group, respectively, were collected prospectively for microbiota analysis after the launch of this study in July 2017 (Fig. 1). Clinical data, including demographic information, comorbidities, clinical presentations, laboratory parameters, and recent medications within 28 days of inclusion, were obtained from medical records. Patients with major burns, trauma, surgery, severe pancreatitis, cerebral vascular accident, acute organ failure, or admission to an intensive care unit within 4 weeks before CDI onset met the definition of current critical illness status. In addition, the crude mortality rate was recorded. These patients were monitored until December 2019, with a mean follow-up period of 360.7 ± 341.3 days. This study was approved by the Chang Gung Medical Foundation Institutional Review Board (approval code 201601449A3).

### Sample collection, DNA extraction, and amplicon library construction.

Fresh stool samples (approximately 3 g) from 79 CDI patients and 66 non-CDI patients were collected in stool collection containers (OMNIgene.GUT OMR-200 kit), immediately frozen, and/or transported with ice packs to the laboratory within 4 h after collection. The samples were stored at −80°C until DNA extraction. The extraction of genomic DNA from 200 mg of the fecal sample was carried out using the commercial QIAmp Fast DNA Stool minikit (Qiagen, Germany) according to the manufacturer's instructions. The PCR and 16S rRNA gene sequencing library protocols were performed at the University of Michigan Host Microbiome Core as described previously by Koenigsknecht et al. (43) and the International Human Microbiome Standards (IHMS) project (http://www.microbiome-standards.org). The V4 region of the 16S rRNA gene was amplified from each sample using a dual-indexing sequencing strategy, and the amplicons were sequenced on the Illumina MiSeq platform, as also described previously by Koenigsknecht et al. (43).

### Bioinformatic analysis.

The USEARCH tool v11 (https://drive5.com) for amplicon sequence variant (ASV) inference was used to process the 16S rRNA gene amplicon sequencing reads, and the taxonomic assignment was generated by using the SINTAX classifier (44). The diversity and species richness of fecal microbiota were determined from the number of bacterial species assigned by the ASVs. Linear discriminant analysis effect size (LEfSe) (45, 46) was used to identify the taxonomy of fecal bacteria that were most likely to distinguish between cases of PPI user and nonuser and between cases of survivor and victim.

### Statistical analysis.

The clinical manifestations, recent medical insults, and initial biochemical data for the included patients were introduced to calculate descriptive statistics and compared with nonparametric tests using SPSS statistical software v20.0 (IBM, Armonk, NY, USA). Continuous variables are described as mean with standard deviation (SD). Categorical variables are expressed as numbers and percentages. Kaplan-Meier curves estimated the survival times with a log rank test for statistical comparison. Cox regression analyses were used to identify the hazard factors associated with death. The relative abundance of each ASV was normalized by total sum scaling (TSS), which is usually utilized by LEfSe (45). The differences in the relative abundance of each ASV among the subgroups of PPI users were analyzed using the Mann-Whitney U test. Each ASV was log transformed for the survival analyses to achieve linearity. The Kaplan-Meier survival analyses and Cox regression analyses were performed with MATLAB and Statistics Toolbox Release 2018b (MathWorks, Inc., Natick, MA, USA). *P* values of <0.05 were regarded as statistically significant.

### Data availability.

The sequence data have been deposited in the NCBI Sequence Read Archive (SRA) under the BioProject accession number PRJNA760324.
