# Supplementary material for: Proton Pump Inhibitor-Induced Gut Dysbiosis Increases Mortality Rates for Patients with Clostridioides difficile Infection
Source: Microbiol Spectr. 2022 Jul 6;10(4):e00486-22. doi: 10.1128/spectrum.00486-22 (PMC9430933; doi:10.1128/spectrum.00486-22)
Supplement: Supplemental file 1 — Supplemental material. Download spectrum.00486-22-s0001.pdf, PDF file, 0.1 MB [file spectrum.00486-22-s0001.pdf]

Supplemental Tables

Table S1. Cox regression analyses for the hazard factors associated with mortality within 180 days between proton pump inhibitor use and comorbidities.

	Univariate		Multivariate			
	HR	p values	HR	95% CI lower	95% CI upper	p values
PPI (per week)	1.287	<0.01	1.228	1.077	1.402	<0.01
Diabetes	1.063	0.77				
Diabetes with end organ damage	0.927	0.75				
Chronic kidney disease	1.852	<0.01	2.296	1.455	3.624	<0.01
Hematological cancer	2.286	<0.01	3.474	2.013	5.996	<0.01
Solid organ cancer	2.895	<0.01	1.65	0.88	3.095	0.12
Metastatic solid organ cancer	3.726	0	3.022	1.519	6.011	<0.01
Old cerebral vascular accident	1.047	0.87				
Connective tissue disorder	0.21	0.03	0.374	0.091	1.542	0.17
Liver disease	1.107	0.68				
Portal hypertension	1.757	0.18				
Hemiplegia	0.61	0.14				
Peripheral arterial occlusive disease	1.932	0.05	2.823	1.426	5.589	<0.01
History of Myocardial infarction	0.644	0.3				
Dementia	0.569	0.22				
Congestive heart failure	1.229	0.51				
Chronic obstructive pulmonary disease	2.403	<0.01	2.349	1.247	4.422	<0.01
Recent peptic ulcer disease*	1.216	0.58				

* Endoscopic proven peptic ulcer disease within 3 months ahead of index day.

Table S2. The gut microbes significantly changed their relative abundance among CDI patients with vs. without PPI use. The relative abundance of ASV in CDI patients without PPI use defined as a baseline group (relative abundance < 0.1% included, p value > 0.05 excluded).

Any PPI use vs. No PPI use	LDA Score	p value
Parabacteroides merdae	4.32	0.05
Odoribacter splanchnicus	3.28	0.05
Xanthomonadales order	2.72	0.04
Stenotrophomonas	2.68	0.04
Xanthomonadaceae family	2.54	0.04
Bifidobacterium bifidum	2.08	0.04
Peptostreptococcus stomatis	-2.42	0.01
Fusicatenibacter	-2.46	< 0.01
Terrisporobacter	-2.74	< 0.01
Terrisporobacter mayombei	-2.75	< 0.01
Clostridium disporicum	-2.94	0.03
Peptostreptococcus	-3.29	< 0.01
Bifidobacteriaceae family	-3.41	0.03
Bifidobacterium	-3.41	0.02
Bifidobacteriales order	-3.42	0.03
Actinobacteria class	-3.53	0.05
Actinobacteria phylum	-3.53	0.05
Comamonadaceae family	-3.57	0.03
Comamonas	-3.63	0.03
Prevotella copri	-3.80	0.04
Betaproteobacteria class	-4.03	0.05
Veillonella dispar	-4.12	0.02
Veillonella	-4.16	0.02

ASV, amplicon sequence variants; CDI, *Clostridioides difficile* infection; LDA, Linear discriminant analysis; PPI, proton pump inhibitor

Table S3. The changes in gut microbiota after PPI ≥ 28 days among CDI patients (relative abundance < 0.1% included, p value > 0.05 excluded)

In prolonged (≥28 days) PPI use vs. No PPI use	LDA Score	p value
Bacteroides pyogenes	3.85	0.03
Bacteroides cellulosilyticus	3.24	0.04
Peptoniphilus	2.97	0.04
Peptoniphilaceae family	2.97	0.04
Peptoniphilus duerdenii	2.93	0.03
Anaerostipes rhamnosivorans	2.85	0.03
Propionispira	2.60	0.03
Clostridium lactatifermentans	-2.71	0.03
Lachnospiracea incertae sedis	-3.42	0.01
Ruminococcus gnavus	-3.44	0.04
Betaproteobacteria class	-4.19	0.05

CDI, *Clostridioides difficile* infection; LDA, Linear discriminant analysis; PPI, proton pump inhibitor

Table S4. The coefficient analysis between *C. difficile* and other target bacteria

Spearman's rho coefficient (p)	*P. Merdae* abundance	*P. copri* abundance	*R. gnavus* abundance
C. difficile abundance	0.057<br>-0.62	-.315** -0.005	-0.19<br>-0.094

** $p < 0.01$

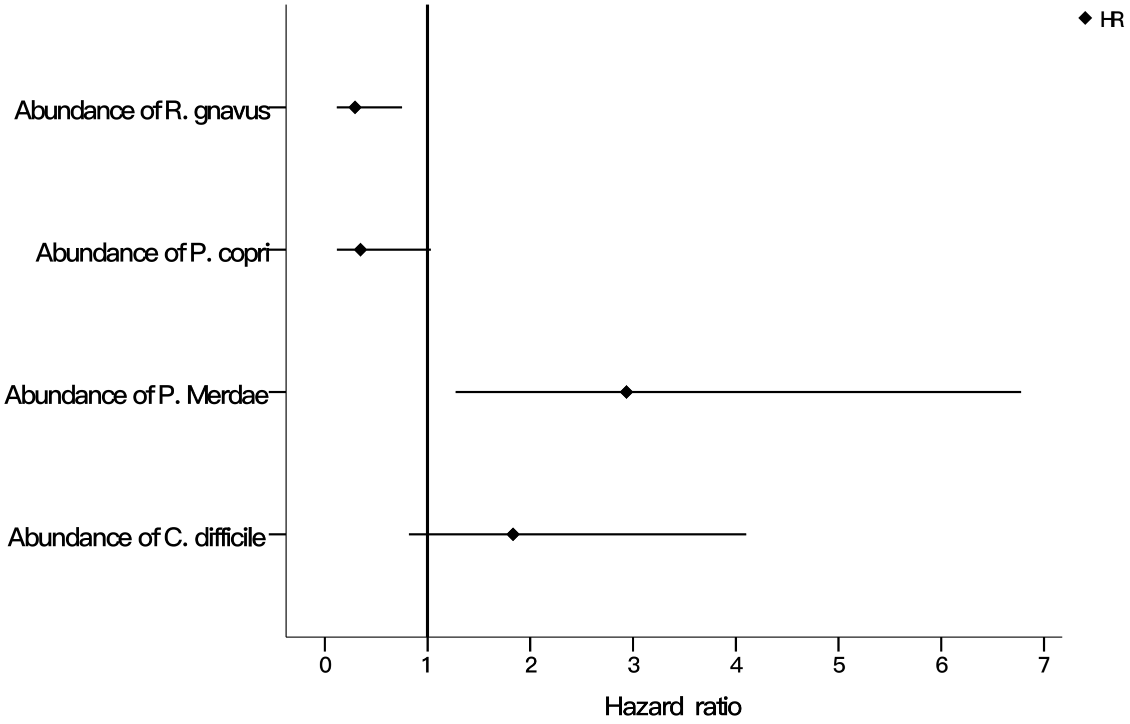

Figure S1. Forest plot for multivariate Cox regression for the relative abundance of each ASV on the mortality rate at day 180 of CDI patients.
